# Applying the 2024-revised McDonald criteria for multiple sclerosis using conventional diagnostic tools: a single-centre prospective cohort study in Germany

**DOI:** 10.1016/j.eclinm.2026.104098

**Published:** 2026-07-25

**Authors:** Franz Felix Konen, Nima Mahmoudi, Lina Marie Albers, Georgina Arrambide, Anna Lena Streichert, Melanie Haar, Alexander Soldatov, Erda Bucak, Konstantin Fritz Jendretzky, Sandra Nay, Lea Grote-Levi, Tobias Hegelmaier, Corinna Trebst, Philipp Schwenkenbecher, Martin W. Hümmert, Marc Pawlitzki, Kurt-Wolfram Sühs, Michael Khalil, Carsten Framme, Axel Petzold, Sven G. Meuth, Mike P. Wattjes, Aiden Haghikia, Xavier Montalban, Julius Renne, Thomas Skripuletz

**Affiliations:** a Department of Neurology, Hannover Medical School, Carl-Neuberg-Str. 1, 30625 Hannover, Germany; b Department of Neuroradiology, Charité Universitätsmedizin Berlin, Corporate Member of Freie Universität Berlin, Humboldt-Universität Zu Berlin, Charitéplatz 1, 10117 Berlin, Germany; c Neurology-Neuroimmunology Department, Vall d'Hebron Barcelona Hospital Campus, Universitat Autònoma de Barcelona, and Universitat de Vic-Central de Catalunya (UVic-UCC); Barcelona, Spain; d Department of Neuroradiology, Hannover Medical School, Carl-Neuberg-Straße 1, Hannover 30625, Germany; e Department of Ophthalmology, Hannover Medical School, Carl-Neuberg-Straße 1, Hannover 30625, Germany; f Department of Neurology, Klinikum Agnes Karll Laatzen/Klinikum Region Hannover, Hildesheimer Str. 158, 30880 Laatzen, Germany; g Department of Neurology, University Hospital and Medical Faculty Düsseldorf, Heinrich Heine University Düsseldorf, 40225 Düsseldorf, Germany; h Department of Neurology, Medical University of Graz, Auenbruggerplatz 22, 8036 Graz, Austria; i The National Hospital for Neurology and Neurosurgery, Queen Square UCL Institute of Neurology & Moorfields Eye Hospital, City Road, London, UK; j Department of Neurology, University of Münster, Münster 48149, Germany

**Keywords:** Multiple sclerosis, McDonald criteria, Cerebrospinal fluid, Visual evoked potentials, MRI

## Abstract

**Background:**

The 2024-revised McDonald criteria may enable earlier diagnosis of multiple sclerosis (MS) through inclusion of optic nerve involvement as fifth neuroanatomical topography, novel MRI biomarkers, cerebrospinal fluid (CSF) kappa free light chains (KFLC), and classification of radiologically isolated syndrome (RIS) as MS. Their applicability in routine practice remained for evaluation.

**Methods:**

In this prospective single-centre cohort study in Germany, adults presenting between October 1, 2020, and June 30, 2025, with a first demyelinating event or RIS were screened. Participants underwent conventional diagnostic work-up according to the 2017-revised McDonald criteria, including contrast-enhanced MRI, visual evoked potentials (VEP), and CSF analysis. Novel MRI markers and optical coherence tomography were not systematically available. Primary outcome was the proportion of individuals fulfilling the 2024-revised versus the 2017-revised McDonald criteria at baseline assessment.

**Findings:**

Applying the 2024-revised criteria, 201/215 individuals (93.5%) fulfilled diagnostic criteria for MS versus 181/215 (84%) using the 2017 revision (p < 0.0001). Additional diagnoses resulted from optic nerve involvement (55%) and RIS reclassification (35%). VEP identified optic nerve lesions in 89% of cases. Overall, 102/201 (51%) individuals fulfilled diagnostic criteria based on MRI findings alone, whereas 99/201 (49%) additionally required inflammatory CSF findings and dissemination in time.

**Interpretation:**

Application of selected components of the 2024-revised McDonald criteria increased the proportion of individuals fulfilling the diagnostic criteria, mainly through inclusion of optic nerve involvement and recognition of RIS. CSF analysis remained important for diagnosis and differential diagnostics. Further studies should evaluate the contribution of novel imaging biomarkers.

**Funding:**

No external funding was received.

**Research in context**

**Evidence before this study**

While the diagnosis of multiple sclerosis (MS) was established by demonstrating dissemination in space (DIS) and time (DIT) under earlier versions of the McDonald criteria, the criteria were revised in 2024 to broaden the framework and facilitate earlier diagnosis. The updated criteria introduce several modifications, including recognition of optic nerve involvement as an additional neuroanatomical topography for the demonstration of DIS and the incorporation of emerging biomarkers and different tools such as the central vein sign (CVS), paramagnetic rim lesions (PRL), optical coherence tomography (OCT), and kappa free light chains (KFLC) in cerebrospinal fluid (CSF). Although these changes are intended to increase diagnostic sensitivity and enable earlier identification of MS, concerns have been raised regarding their implementation in routine clinical practice, particularly in settings where advanced imaging techniques are not widely available and regarding the potential risk of overdiagnosis in individuals with radiologically isolated syndrome (RIS).

To assess the available evidence before undertaking this study, we searched PubMed/MEDLINE and Web of Science from database inception to Feb 1, 2026, using the terms (“multiple sclerosis” AND “McDonald criteria” AND (”2024” OR “revision” OR “diagnostic criteria”)) combined with terms related to “radiologically isolated syndrome”, “central vein sign”, “paramagnetic rim lesions”, “optical coherence tomography”, and “kappa free light chains”. We also screened reference lists of relevant articles and reviews. Available evidence mainly consists of expert consensus papers describing the proposed criteria and studies evaluating individual biomarkers, whereas prospective real-world studies assessing the overall diagnostic impact of the revised criteria remain scarce. In particular, the extent to which the revised framework increases diagnostic yield when applied using conventional diagnostic work-up has not yet been systematically evaluated.

**Added value of this study**

In this prospective cohort study, we evaluated the applicability of the 2024-revised McDonald criteria using conventional diagnostic work-up routinely employed in clinical practice, including contrast-enhanced MRI, CSF, and VEP. By not accounting for newly introduced markers such as CVS, PRL, and OCT, we assessed the diagnostic impact of the revised framework using already established diagnostic parameters. Our findings show that the 2024 revision substantially increases the proportion of patients diagnosed with MS at the time of the first clinical event compared with the 2017 criteria. Furthermore, we provide detailed insights into the relative diagnostic contribution of different components of the revised criteria and demonstrate the central role of CSF and optic nerve involvement for early diagnosis.

**Implications of all the available evidence**

Together with existing evidence, our findings suggest that the 2024 revision of the McDonald criteria may facilitate earlier diagnosis of MS in routine clinical practice, even in settings without availability of additional imaging markers such as CVS and PRL. Conventional diagnostic tools including MRI, CSF, and VEP remain highly informative and allow most diagnoses to be established without the need for novel imaging markers. These results support a pragmatic implementation of the revised criteria in real-world clinical settings while highlighting the need for further studies validating the role of emerging biomarkers and evaluating their impact on diagnostic accuracy and potential risks of overdiagnosis.

## Introduction

Multiple sclerosis (MS) is the most frequent chronic, autoimmune-mediated demyelinating disorder of the central nervous system (CNS), predominantly affecting young adults.^1^^,^^2^ The therapeutic landscape has evolved considerably over the past two decades, with multiple disease-modifying therapies (DMT) capable of reducing relapse rates, attenuating disease activity, and delaying disability progression.^3^^,^^4^ Early and accurate diagnosis is critical to optimise treatment efficacy, thus reducing neuronal damage and preventing neurological disability.^5^^,^^6^ To standardise and expedite diagnostic pathways, the McDonald criteria for diagnosing MS have been established, integrating clinical, magnetic resonance imaging (MRI evidence to demonstrate dissemination in space (DIS) and time (DIT)), and cerebrospinal fluid (CSF) evidence to confirm the inflammatory nature of the symptoms and MRI findings supporting a more biological-based diagnosis.^7^

Recently the new 2024 revision of the McDonald diagnostic criteria together with their companion papers were published following expert panel discussions and revisions.^8^, ^9^, ^10^, ^11^ The 2024 revision introduces substantial modifications that reflect advances in neuroimaging, neurophysiological assessment, and biomarker discovery.^8^ The optic nerve is incorporated as the fifth neuroanatomical topography for establishing DIS, complementing periventricular, (juxta-)cortical, infratentorial, and spinal cord regions.^8^, ^9^, ^10^ Optic nerve involvement can be substantiated by visual evoked potentials (VEP), optical coherence tomography (OCT), or MRI thus formally integrating visual pathway assessment into the diagnostic framework.^8^, ^9^, ^10^ MS can be diagnosed when ≥4 typical topographies are affected.^8^, ^9^, ^10^ Furthermore, novel supportive biomarkers have been recognised: in MRI, the central vein sign (CVS) and paramagnetic rim lesions (PRL) serve as very specific markers of inflammatory demyelination such as in MS; in CSF, intrathecal inflammation can be demonstrated by oligoclonal bands (OCB) or by kappa free light chains (KFLC), which represent a complementary biomarker.^8^, ^9^, ^10^, ^11^, ^12^ Lastly, the criteria now permit a diagnosis of MS in individuals with incidental MRI findings suggestive of chronic inflammatory demyelination (RIS: radiologically isolated syndrome) and in people with non-specific presentations.^8^^,^^13^ These revisions aim to increase diagnostic sensitivity while incorporating additional features intended to preserve, even increase, diagnostic specificity, thereby enabling an earlier and more accurate diagnosis of MS.^8^ However, the real-world impact and applicability of these newly incorporated modifications on diagnostic decision-making, particularly in heterogeneous clinical presentations, especially compared to the conventional routine diagnostics remains to be fully elucidated. Recent studies have provided initial evidence for the diagnostic performance of individual components of the revised criteria, suggesting high sensitivity and accuracy of certain approaches.^14^ However, overall evidence remains limited and the relative importance of different diagnostic tools within the revised framework has not yet been systematically evaluated.^14^ Particularly, the newly introduced MRI biomarkers are today not yet readily available in all centres and their availability will only increase over time.^9^

Therefore, the objective of the present study was to investigate the application of the revised 2024 McDonald criteria in a real-world setting using conventional and well-established MRI markers and CSF diagnostic methods already available (paraclinical tools as per 2017-revised McDonald criteria) and to assess the influence on diagnoses in patients with suspected demyelinating diseases of the CNS.

## Methods

### Patients

Patients presenting with a first clinical event suggestive of a chronic inflammatory disease of the CNS, as well as asymptomatic individuals with MRI findings compatible with RIS according to established criteria, were prospectively included between October 1, 2020, and June 30, 2025 at the Department of Neurology, Hannover Medical School.^15^^,^^16^ No formal sample size calculation was performed because all consecutive eligible individuals presenting during the study period were included and this study was rather exploratory and hypothesis-generating. In individuals with RIS, the absence of current or prior neurological symptoms suggestive of demyelinating disease was confirmed by structured clinical history and neurological examination. Reasons for the initial MRI leading to identification of RIS included headache (4/8), trauma (2/8), and non-specific fatigue (2/8). Patients were prospectively enrolled and underwent a standardised diagnostic work-up. The application of the 2024-revised McDonald criteria was performed retrospectively as a re-classification analysis using the prospectively acquired clinical, MRI, VEP, and CSF data.

Inclusion criteria were: 1) age ≥18 years, 2) referral for suspected MS due to a first clinical event suggestive of inflammatory demyelinating CNS disease, or incidental MRI findings compatible with RIS, 3) presentation within six months from symptom onset or, in individuals with RIS, within six months from first MRI evidence of demyelinating lesions.

All patients underwent a standardised diagnostic work-up aimed at corroborating subjective symptoms by objective findings with visual impairment required to at least fulfil the criteria for possible optic neuritis according to the ICON (International Consensus on Optic Neuritis) 2022 criteria.^17^ Sex was recorded as biological sex (female or male) from the medical records at study inclusion. Gender identity was not systematically collected.

After completion of the diagnostic work-up, patients were excluded from final analysis, if: 1) no objective evidence supporting a demyelinating CNS disorder was identified, 2) detailed history revealed prior neurological events suggestive of chronic inflammatory CNS disease, 3) symptom onset or first MRI evidence preceded presentation at Hannover Medical School and diagnostic work-up by more than six months, or 4) an alternative diagnosis was established, such as neuromyelitis optica spectrum disorders (NMOSD), myelin oligodendrocyte glycoprotein antibody-associated disease (MOGAD), vascular, or neoplastic disease. Loss to follow-up did not lead to exclusion from analysis.

**Fig. 1:**
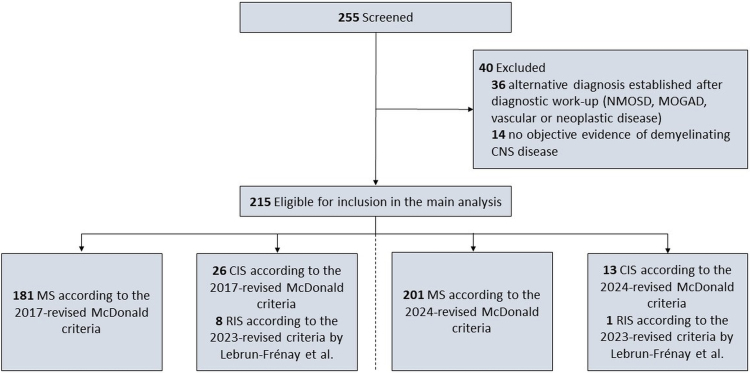
**Study profile.** Screened patients were excluded based on identification of alternative diagnoses or missing objective findings of demyelinating disease of the central nervous system after diagnostic work-up. The eligible 215 patients were diagnosed with multiple sclerosis or clinically isolated syndrome according to the 2017- or 2024-revised McDonald criteria or radiologically isolated syndrome according to the 2023-revised criteria by Lebrun-Frénay et al. MS = multiple sclerosis; CIS = clinically isolated syndrome; RIS = radiologically isolated syndrome; NMOSD = neuromyelitis optica spectrum disease; MOGAD = myelin oligodendrocyte glycoprotein antibody associated disease; CNS = central nervous system.

Patients were prospectively enrolled and underwent a standardised diagnostic work-up. A flowchart of participant selection is shown in Fig. 1.

The primary outcome was the proportion of individuals fulfilling the 2024-revised compared with the 2017-revised McDonald criteria. Secondary outcomes included the relative contribution of optic nerve involvement, inflammatory CSF findings, and RIS reclassification to additional MS diagnoses, as well as exploratory diagnostic scenario analyses evaluating the impact of excluding individual diagnostic components. The primary and secondary outcomes were prespecified in the study protocol. Exploratory diagnostic scenario analyses and theoretical evaluations of additional biomarkers (CVS, PRL, and OCT) were performed post hoc.

### Clinical, paraclinical and laboratory examination

As part of the “conventional routine diagnostic work-up” in accordance with the 2017-revised McDonald criteria and the German guideline for treatment and diagnosis of MS, the following clinical, paraclinical and laboratory analyses were performed in all patients: clinical examination, VEP (pathological findings defined as P100 latency >120 ms or absence of a measurable P100 response), SEP, contrast-enhanced (single-dose of gadolinium-based contrast media) MRI of the brain and spinal cord (acquisition at Hannover Medical School), CSF routine analysis to assess for inflammatory changes.^10^^,^^18^, ^19^, ^20^

Complete neurological examination was performed by experienced board-certified neurologists. Laboratory analyses included testing for ANA (antinuclear antibodies), ENA (extractable nuclear antigens), ANCA (anti-neutrophil cytoplasmic antibodies), lues, and Borrelia in all patients, and for aquaporin-4 and MOG antibodies in patients with optic neuritis or myelitis, to exclude inflammatory, infectious, and rheumatological differential diagnoses. Antibodies against MOG and aquaporin-4 were only tested, when diagnosis of MS seemed uncertain, especially in the case of “atypic” optic neuritis or isolated or non-MS-typical myelitis at first sight. As shown in Fig. 1, some patients were afterwards excluded due to other final diagnoses.

Cerebral and spinal MRI was performed at Hannover Medical School on a 3 T whole-body MR system (Skyra, Siemens Healthineers, Erlangen, Germany) using dedicated head and spine coils using the same standardised MRI protocol as previously described.^21^^,^^22^ MRI acquisition followed the 2021 MAGNIMS-CMSC-NAIMS guidelines and included standard-dose gadolinium enhancement for detection of active inflammatory lesions.^21^^,^^22^

CSF and serum analyses for all participants were conducted at the Neurochemistry Laboratory, Department of Neurology, Hannover Medical School as described previously.^22^ Concentrations of immunoglobulins (Ig) G, A, M, and albumin in both CSF and serum were measured by kinetic nephelometry (IMMAGE system, Beckman Coulter, Brea, CA, USA). Intrathecal synthesis of each immunoglobulin class was calculated according to the method described by Reiber.^23^ CSF-specific OCB were detected by isoelectric focussing on polyacrylamide gels (EDC, Tübingen, Germany), followed by silver staining.^24^ Two isolated CSF-bands were interpreted as evidence of intrathecal immunoglobulin production.^12^^,^^24^

KFLC were measured in accordance with the methods provided by the manufacturer using the N Latex FLC Kappa Kit (Siemens Healthineers, Marburg, Germany) on a Neph Atellica 630 System (Siemens). As cut-off, the recently proposed KFLC index ((CSF KFLC concentration/serum KFLC concentration)/(CSF albumin concentration/serum albumin concentration)) of 6.1 was used.^11^^,^^12^^,^^25^ KFLC were measured as part of the initial diagnostic work-up at the time of patient presentation and before final diagnostic classification.

### Diagnostic scenario analyses: role of VEP and additional criteria

As all patients underwent the full conventional diagnostic work-up, additional analyses were performed to model hypothetical diagnostic scenarios by selectively excluding individual diagnostic components. By selectively excluding individual diagnostic components (e.g., VEP, CSF findings, or gadolinium-enhancing lesions), the incremental diagnostic value of additional criteria introduced in the 2024-revised McDonald criteria and their impact on the proportion of patients fulfilling the diagnostic criteria for MS was assessed. Thus, using the prospectively collected data, patients were retrospectively re-classified according to the 2024-revised McDonald criteria. These analyses can be considered sensitivity analyses exploring the robustness of diagnostic classification when individual diagnostic components were selectively excluded.

In addition, exploratory analyses were performed in patients classified as clinically isolated syndrome (CIS) or RIS. These analyses evaluated whether the application of additional imaging or paraclinical markers proposed in the revised criteria (CVS, PRL, or OCT) could theoretically allow a diagnosis of MS. These exploratory post-hoc analyses assessed the potential contribution of individual diagnostic components to the 2024-revised McDonald criteria and should be interpreted as hypothetical scenarios that do not imply the actual presence of such markers in all cases.

### Ethics

The study was conducted in accordance with the Declaration of Helsinki and its subsequent revisions. Ethical approval was obtained from the Ethics Committee of Hannover Medical School (MHH), Hannover, Germany (No. 8819_BO_S_2019). Written informed consent for study participation, collection of clinical and paraclinical data, and their use for scientific analyses and publication was obtained from all participants. The study was conducted according to a prespecified study protocol, which is provided as a Supplementary Appendix. As the study was initiated and conducted in Germany, the original study protocol is provided in German. All data were anonymised before analysis. This study followed the Strengthening the Reporting of Observational Studies in Epidemiology (STROBE) reporting guideline.

### Statistical analysis

Normality of continuous variables was assessed visually and using the D'Agostino & Pearson omnibus normality test to determine whether parametric or non-parametric statistical methods were appropriate. Variables with a normal distribution were summarised as mean values, while non-normally distributed variables were presented as medians, each accompanied by their respective standard deviation or interquartile range (IQR). Comparisons between independent groups were conducted using the Wilcoxon rank-sum test for continuous data and the Chi-squared test for categorical data. For paired observations, the paired t-test was applied when data were Gaussian, whereas the Wilcoxon signed-rank test was used for non-Gaussian distributions. Differences in paired diagnostic classifications between the 2017- and 2024-revised McDonald criteria were assessed using McNemar's exact test, as both diagnostic frameworks were applied to the same individuals. All statistical analyses and figures were generated with SPSS version 28.0 (IBM, Armonk, NY, USA) and GraphPad Prism version 8.4.3 (GraphPad Software, La Jolla, CA, USA).

### Role of the funding source

No funding was received for this work.

## Results

Of the included patients, 135/215 (63%) were female, with a median age at diagnosis of 34 years (IQR: 27–45). None of the patients included received DMT at the time of study inclusion. All participants underwent spinal cord MRI and spinal cord lesions were identified in 140/215 (65%) individuals, including 20/215 (9%) with gadolinium-enhancing lesions. Clinical spinal cord symptoms were present in 47 patients (41/47 with spinal cord symptoms only, 6/47 polysymptomatic including spinal cord symptoms), indicating that a substantial proportion of spinal cord lesions were clinically asymptomatic. No baseline data required for diagnostic classification were missing. Follow-up data were available in 116/215 (54%) participants. The baseline data are summarised in Table 1.

**Table 1:** Demographic and clinical characteristics.

Characteristic	All patients (n = 215)	Patients with neurological symptoms (CIS, MS) (n = 207)	Asymptomatic patients (RIS) (n = 8)
Women, n (%)	135 (63%)	131 (63%)	4 (50%)
Men, n (%)	80 (37%)	76 (37%)	4 (50%)
Time from symptom onset or first incidental MRI findings compatible with radiologically isolated syndrome to diagnosis [days], median (IQR)	18 (10–50)	18 (10–42)	14 (9–131)
Age at diagnosis [years], median (IQR)	34 (27–45)	34 (28–45)	36 (25–46)
Multiple sclerosis according to 2024-revised McDonald criteria, n (%)	201 (93%)	194 (94%)	7 (88%)
No multiple sclerosis according to 2024-revised McDonald criteria (clinically isolated syndrome), n (%)	13 (6%)	13 (6%)	0
No multiple sclerosis according to 2024-revised McDonald criteria (radiologically isolated syndrome), n (%)	1 (0.5)	0	1 (12)
Clinical presentation			
Optic neuritis, n (%)	76 (35%)	76 (37%)	n/a
Spinal cord symptoms, n (%)	41 (19%)	41 (20%)	n/a
Brainstem symptoms, n (%)	30 (14%)	30 (14%)	n/a
Isolated sensory symptoms, n (%)^a^	30 (14%)	30 (14%)	n/a
Isolated motor symptoms, n (%)^a^	18 (8%)	18 (9%)	n/a
Polysymptomatic, n (%)	12 (6%)	12 (6%)	n/a
EDSS, median (IQR)	2 (1–3)	2 (1–3)	0

EDSS = expanded disability status scale; CIS = clinically isolated syndrome; MS = multiple sclerosis; RIS = radiologically isolated syndrome; MRI = magnetic resonance imaging; IQR = interquartile range; n/a = not applicable.

a Symptoms not suggestive for myelitis and no evidence of spinal cord lesions.

Applying the 2017-revised McDonald criteria after initial diagnostic work-up, 181/215 (84%) of the included patients were diagnosed with MS, whereas 26/215 (12%) remained as CIS and 8/215 (4%) as RIS. Among patients presenting with a first demyelinating event, 181/207 (87%) fulfilled the 2017-revised McDonald criteria for MS, whereas 26/207 (13%) remained classified as CIS. By definition, none of the individuals with RIS could be diagnosed with MS according to the 2017-revised criteria. Over a median follow-up of 12 months (IQR 9–16), data were available from 22/34 (65%) patients without an initial diagnosis of MS. During follow-up, 4/16 (25%) patients with CIS and 2/6 (33%) individuals with RIS fulfilled the 2017-revised McDonald criteria for MS. According to the 2024-revised McDonald criteria 3/4 patients with CIS and 2/2 individuals with RIS could have been diagnosed with MS after initial diagnostic work-up, leading to a median diagnostic delay of 16 months for CIS (IQR: 10–19) and 11 months for individuals with RIS (IQR: 9–13).

**Fig. 2:**
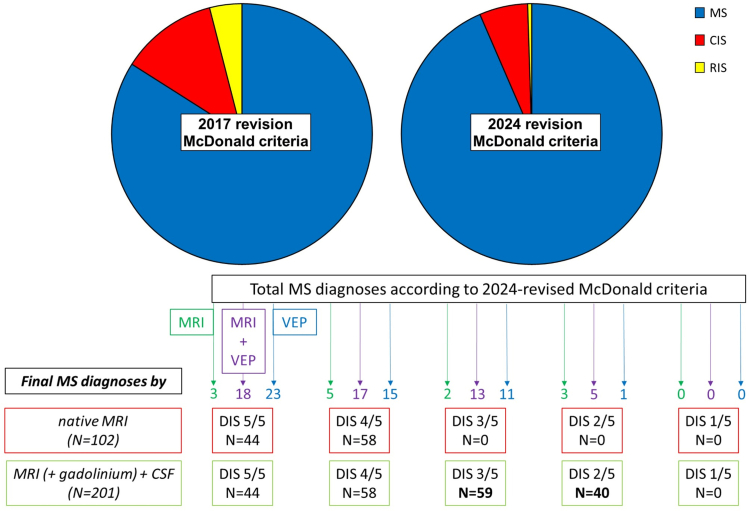
**Multiple sclerosis (MS), clinically and radiologically isolated syndromes (CIS, RIS) according to 2017- and 2024-revised McDonald criteria.** The pie charts depict the proportion of patients with multiple sclerosis (MS, blue), clinically isolated syndrome (CIS, red), and radiologically isolated syndrome (RIS, yellow) according to the 2017- and 2024-revised McDonald criteria. The flow diagram illustrates how MS diagnoses according to the 2024-revised criteria were established based on dissemination in space (DIS), indicating the number of affected neuroanatomical topographies (e.g. 5/5), and the contribution of optic nerve involvement demonstrated by MRI (green), visual evoked potentials (VEP, blue), or both (purple). It further distinguishes MS diagnoses based on native MRI findings alone (red) from those requiring additional diagnostic features, including gadolinium-enhancing lesions and cerebrospinal fluid (CSF) findings (light green). The flow diagram highlights that the increase in MS diagnoses under the 2024-revised criteria is primarily driven by incorporation of optic nerve involvement and by allowing diagnosis in patients with limited DIS when supported by additional diagnostic features. Differences between paired diagnostic classifications were assessed using McNemar's exact test. DIS = dissemination in space; VEP = visual evoked potentials; MRI = magnetic resonance imaging; CSF = cerebrospinal fluid.

Applying the latest revision of the McDonald criteria for MS of 2024 after initial diagnostic work-up, 93.5% of the patients (201/215) were diagnosed with MS, whereas 6% (13/215) remained as clinically isolated syndrome (CIS) and 0.5% (1/215) as RIS (Fig. 2) when conventional routine diagnostics only were used (compared to 2017-revised McDonald criteria p < 0.0001, McNemar's exact test). Among patients presenting with a first demyelinating event, 194/207 (94%) fulfilled the 2024-revised McDonald criteria for MS, whereas 13/207 (6%) remained classified as CIS (compared with 2017-revised McDonald criteria p = 0.0002). Among the 8 individuals with RIS, 7 (88%) fulfilled the 2024-revised criteria for MS and 1 (12%) remained classified as RIS (compared to 2017-revised McDonald criteria p = 0.016). On the initial conventional MRI, 102/201 (51%) patients fulfilled the diagnostic criteria based on dissemination in space (DIS) alone (≥4/5 neuroanatomical topographies), without the need of other findings. An additional 99/201 (49%) patients fulfilled the 2024 McDonald criteria with DIS in 2–3 neuroanatomical topographies in combination with additional diagnostic features, namely dissemination in time (DIT) demonstrated by gadolinium-enhancing lesions and/or CSF-specific oligoclonal bands or intrathecal kappa free light chain synthesis (46/99 with DIT and positive CSF findings; 50/99 with positive CSF findings without DIT; 3/99 with DIT but without inflammatory CSF findings).

Of the 20 additional MS diagnoses according to the 2024-revised McDonald criteria compared with the 2017 revised McDonald criteria, 65% (13/20) derived from patients with CIS, which were mainly diagnosed (11/13, 85%) due to consideration of the optic nerve as fifth neuroanatomical topography (Table 2). Involvement of the optic nerve was demonstrated with MRI and VEP in 6/11, VEP only in 1/11 or MRI only in 4/11. 2/13 (15%) patients with CIS could have been diagnosed with MS according to the 2024-revised McDonald criteria by demonstration of DIS in ≥4/5 neuroanatomical topographies. 35% (7/20) of the additional MS diagnoses according to the 2024-revised McDonald criteria derived from consideration of individuals with RIS (Table 2). Consideration of inflammatory CSF (CSF-restricted oligoclonal bands and KFLC index) as additional diagnostic feature led to the final MS diagnosis in all individuals with RIS.

**Table 2:** Comparing 2024- and 2017-revised McDonald criteria: additional MS diagnoses.

Additional MS diagnosis due to	n = 20
Consideration of optic nerve as fifth topography for DIS in CIS, n (%)	11/20 (55%)
Thereof detection of optic nerve involvement by MRI and VEP, n (%)	6/11 (55%)^a^
Thereof detection of optic nerve involvement by MRI only, n (%)	4/11 (36%)^b^
Thereof detection of optic nerve involvement by VEP only, n (%)	1/11 (9%)^b^
Diagnosis of MS by MRI only (≥4/5 neuroanatomical topographies) in CIS, n (%)	2/20 (10%)
Thereof fulfilment of DIS with 4/5 topographies by including optic nerve, n (%)	2/2 (100%)^c^
Diagnosis of MS in RIS patients, n (%)	7/20 (35%)
Thereof MS diagnosis by inflammatory CSF (2/5 or 3/5 neuroanatomical topographies), n (%)	7/7 (100%)^b^

MS = multiple sclerosis; CIS = clinically isolated syndrome; RIS = radiologically isolated syndrome; MRI = magnetic resonance imaging; DIS = dissemination in space; DIT = dissemination in time; VEP = visual evoked potentials; CSF = cerebrospinal fluid.

a All with DIT (gadolinium-enhancing lesions) and inflammatory CSF.

b All without DIT but inflammatory CSF.

c All without DIT or inflammatory CSF.

**Fig. 3:**
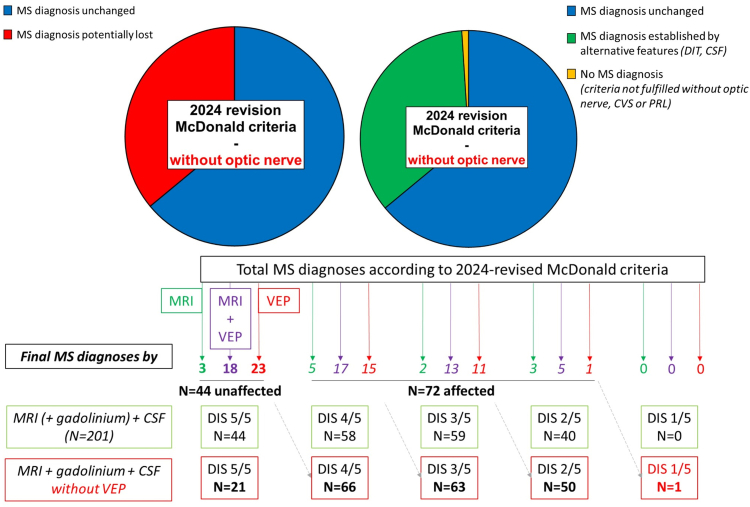
**Multiple sclerosis (MS) diagnoses according to 2024-revised McDonald criteria with hypothetical exclusion of visual evoked potential (VEP) results.** The pie charts illustrate how multiple sclerosis (MS) diagnoses would change if demonstration of optic nerve involvement by visual evoked potentials (VEP) would be hypothetically excluded as a fifth neuroanatomical topography. The left pie chart shows MS diagnoses that would remain unchanged without VEP (blue) compared with those that would require additional diagnostic criteria and would potentially be lost (red). The right pie chart further differentiates MS diagnoses that could still be established based on dissemination in time (DIT) or other additional diagnostic features (green) from diagnoses that would not be possible without optic nerve or novel imaging markers (orange). The flow diagram details the distribution of MS diagnoses according to dissemination in space (DIS; number of affected neuroanatomical topographies) and illustrates how optic nerve involvement was originally demonstrated by MRI (green), VEP (red; hypothetically excluded), or both (purple). It further depicts how MS diagnoses would shift across DIS categories when optic nerve involvement by VEP is not considered (grey arrows). Bold numbers in red boxes indicate MS diagnoses remaining possible without VEP. DIS = dissemination in space; DIT = dissemination in time; MRI = magnetic resonance imaging; VEP = visual evoked potentials; CVS = central vein sign; PRL = paramagnetic rim lesion.

To model a diagnostic scenario without VEP assessment, analyses were repeated after excluding VEP findings used to demonstrate optic nerve involvement according to the 2024-revised McDonald criteria (Fig. 3).

Optic nerve involvement was present in 116/201 patients with MS (58%) and was predominantly identified by VEP (103/116, 89%). When optic nerve involvement as a fifth neuroanatomical topography was excluded (e.g. in the absence of VEP), MS diagnosis remained possible in 129/201 patients (64%). This group comprised 44/129 patients fulfilling DIS in 5/5 neuroanatomical topographies by MRI only and 85/129 patients without evidence of optic nerve involvement on either VEP or MRI. Among the remaining 72/201 patients (36%), MS diagnosis could still be established in 71/72 patients (99%) by fulfilling additional diagnostic criteria, namely DIT demonstrated by gadolinium-enhancing lesions and evidence of intrathecal inflammation by OCB or intrathecal KFLC in patients with DIS in 3/5 or 2/5 neuroanatomical topographies (DIT only: 0/72; DIT and inflammatory CSF: 22/72; inflammatory CSF only: 50/72).

In the remaining patient without DIS, MS diagnosis would require the application of novel imaging markers (CVS or PRL) or pathological OCT findings in combination with additional diagnostic features.

**Fig. 4:**
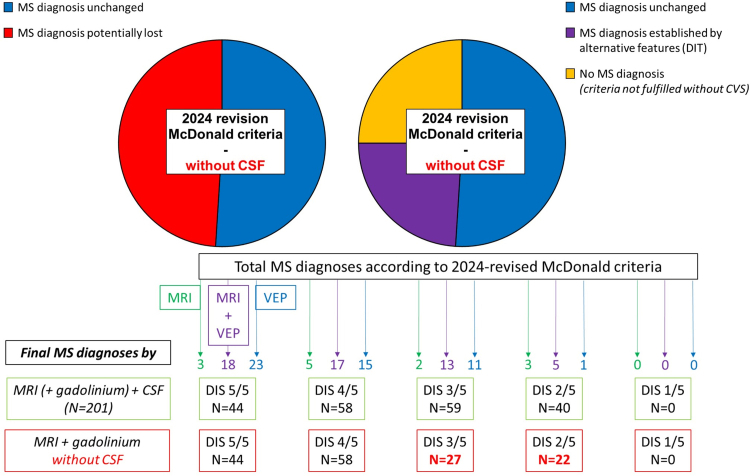
**Multiple sclerosis (MS) diagnoses according to 2024-revised McDonald criteria with hypothetical exclusion of cerebrospinal fluid (CSF) results.** The pie charts illustrate how multiple sclerosis (MS) diagnoses would change if cerebrospinal fluid (CSF) results were not considered as an additional diagnostic criterion. The left pie chart shows MS diagnoses that would remain unchanged without CSF (blue) compared with those that would be affected by exclusion of CSF findings and would be potentially lost (red). The right pie chart further differentiates MS diagnoses that could still be established based on dissemination in time (DIT, purple) from diagnoses that would not be possible without application of novel MRI markers (orange). The flow diagram depicts the distribution of MS diagnoses according to dissemination in space (DIS; number of affected neuroanatomical topographies) and illustrates how optic nerve involvement was originally demonstrated by MRI (green), VEP (blue), or both (purple). It is further illustrated how exclusion of CSF findings affects final MS diagnoses across DIS categories (red boxes). DIS = dissemination in space; DIT = dissemination in time; MRI = magnetic resonance imaging; VEP = visual evoked potentials; CSF = cerebrospinal fluid; CVS = central vein sign.

To model a diagnostic scenario without CSF assessment, analyses were repeated after excluding CSF results as additional diagnostic criteria of the 2024-revised McDonald criteria (Fig. 4).

Under this assumption, 99/201 MS diagnoses (49%) would be affected, whereas 102/201 (51%) diagnoses would remain unchanged due to DIS in ≥4/5 neuroanatomical topographies. Among the 99/201 affected cases, DIT could still be demonstrated by gadolinium-enhancing lesions in 49/99 patients (49%). Consequently, 151/201 patients (75%) would still fulfil the 2024-revised McDonald criteria in the hypothetical absence of CSF results. The remaining missed MS diagnoses comprised patients with DIS in 3/5 (32/50, 64%) and 2/5 (18/50, 36%) neuroanatomical topographies. In this subgroup (50/201), evidence of the CVS would have been required to establish an MS diagnosis in the absence of CSF results.

Clinical and demographic characteristics of the 14 individuals classified as CIS or RIS are shown in Table 1. During a median follow-up of nine months (IQR: 6–13), available in 12/14 patients (86%; two lost to follow-up), three patients were diagnosed with definite MS according to the 2017-revised McDonald criteria due to clinical relapses accompanied by new demyelinating lesions on MRI.

Thirteen patients with neurological symptoms who did not fulfil the diagnostic criteria for MS were classified as CIS, whereas one asymptomatic patient was classified as RIS. Among patients with CIS, 5/13 (38%) demonstrated DIS in 2/5 or 3/5 neuroanatomical topographies but showed neither DIT nor inflammatory CSF findings (assessed in all patients). In this subgroup, the presence of CVS lesions would theoretically allow fulfilment of the diagnostic criteria for MS according to the 2024-revised McDonald criteria. The remaining 8/13 (62%) patients with CIS demonstrated lesions in only one neuroanatomical topography and therefore did not fulfil DIS. In these patients, demonstration of additional markers such as OCT abnormalities consistent with optic nerve involvement or the presence of CVS or PRL could potentially enable an MS diagnosis when combined with other diagnostic features.

## Discussion

In the present study, we assessed the applicability of the proposed 2024-revised McDonald criteria using previously established conventional routine diagnostics, without the consideration of the newly introduced MRI markers (CVS and PRL) as well as OCT.^8^, ^9^, ^10^, ^11^

The main finding of the present study is that application of the routinely available components of the 2024-revised McDonald criteria increases the proportion of patients who can be diagnosed with MS at the time of a first clinical event, even when OCT and novel MRI markers are not considered.^8^ In the presented cohort, 93.5% of patients fulfilled the diagnostic criteria compared to 84% when applying the 2017 revision (McNemar's exact test, p < 0.0001).^7^^,^^8^ This expansion is clinically relevant, as earlier diagnosis enables earlier initiation of DMT, which has been consistently associated with improved long-term outcomes like reduced relapse rates or lower disability accrual.^5^^,^^6^ Nevertheless, a subset presenting with CIS or RIS at baseline remained undiagnosed even with the updated criteria, despite some of them progressing to clinically definite MS during follow-up. On one hand, this finding underscores the continued importance of longitudinal observation in patients not fully meeting diagnostic requirements at first presentation.^13^, ^14^, ^15^, ^16^^,^^26^, ^27^, ^28^ On the other hand, this reflects the importance of validation of diagnostic criteria in real-world settings and shows that despite revising and improving diagnostic criteria, there is still a proportion of patients missed. However, the high rate of MS diagnosis at the first clinical event using conventional diagnostic parameters underscores the additional value of incorporating optic nerve involvement assessed by VEP as well as inflammatory CSF biomarkers (OCB and KFLC) into the revised diagnostic framework.

A key component to the increase in diagnoses was the inclusion of the optic nerve as a fifth neuroanatomical site for DIS.^8^ In the present study, 55% of the additional diagnoses resulted from identifying optic nerve involvement, primarily through VEP. This aligns with recent works that demonstrated that optic nerve lesions have significant diagnostic yield and improve early classification of MS.^22^^,^^29^ Notably, in the present cohort, the second most influential factor was the consideration of asymptomatic individuals with RIS, where diagnosis was typically supported by evidence of an intrathecal synthesis via inflammatory CSF. This is in line with the finding that oligoclonal bands constitute an independent risk factor for conversion of RIS to MS.^30^ In summary, this suggests that the 2024 criteria may capture high-risk RIS cases earlier, potentially influencing surveillance and treatment strategies.^13^, ^14^, ^15^, ^16^^,^^26^, ^27^, ^28^^,^^30^ The possibility of overdiagnosis has nevertheless been raised as a potential concern with the 2024-revised McDonald criteria, particularly in individuals with RIS.^8^^,^^31^ Earlier classification of RIS as MS may carry a risk of misclassification if radiological abnormalities are not truly related to inflammatory demyelination.^30^^,^^31^ In the present cohort, several aspects of the diagnostic work-up likely mitigated this risk. CSF analysis was available in all patients and frequently demonstrated intrathecal inflammation, which is associated with a higher risk of conversion to MS.^13^^,^^15^^,^^30^ In addition, extensive differential diagnostic evaluation and prospective follow-up helped confirm disease evolution in a subset of initially non-diagnostic cases. Together, these findings suggest that the increased diagnostic sensitivity observed in the presented cohort likely reflects earlier identification of patients with a high probability of developing MS rather than substantial overdiagnosis. In line with a recent study reporting high diagnostic sensitivity and accuracy of selected diagnostic approaches within the revised criteria, these findings further support the potential of the 2024 framework to facilitate early diagnosis.^14^ However, while previous work primarily focused on the performance of individual diagnostic components, the present study extends these findings by systematically evaluating the relative contribution and interplay of different conventional diagnostic tools within a real-world clinical setting.^14^ Importantly, the findings presented should be interpreted in the context of the diagnostic components that were available in this cohort. While the 2024-revised McDonald criteria also incorporate novel MRI markers (CVS, PRL) as well as OCT-based assessment of optic nerve involvement, these markers were not systematically acquired and therefore could not be comprehensively evaluated. Consequently, the present study primarily informs the contribution of routinely available diagnostic tools, particularly VEP-based assessment of optic nerve involvement and inflammatory CSF biomarkers including oligoclonal bands and KFLC, to the application of the revised criteria in clinical practice.

The second main finding of the present study is that considering conventional diagnostics only, almost half of the patients presented with DIS in 2/5 or 3/5 neuroanatomical topographies and thus required additional diagnostic features to fulfil the diagnostic criteria for MS.^8^ The remainder could be diagnosed solely on MRI evidence of lesions in ≥4/5 topographies.^8^ None of the diagnosed patients with MS presented without DIS, thus evidence of PRL or CVS was not needed.^8^ The additional diagnostic features compromise demonstration of DIT, evidence of an intrathecal IgG synthesis by oligoclonal bands and/or KFLC and evidence of CVS lesions applying the select-6∗ method.^8^^,^^9^^,^^32^, ^33^, ^34^, ^35^ To investigate the importance of these additional diagnostic features as well as the inclusion of VEP in the diagnostic process, these markers were hypothetically excluded by default. In the present study, hypothetical exclusion of CSF-based evidence as an additional diagnostic feature had the most profound impact, resulting in the largest miss of potential MS diagnoses. In patients with contraindication for CSF analysis, additional MRI sequences for detection of CVS lesions could be useful. When applying the published diagnostic sensitivity of the select-6∗ methods of 82%–89%, hypothetically some of the missed MS diagnoses could have been made, possibly reaching a similar proportion of MS diagnosis as compared with detection of KFLC.^11^^,^^12^^,^^32^^,^^33^ However, CSF analysis in general and detection of CSF-restricted oligoclonal bands in special are pivotal for differential diagnosis beyond the diagnostic contribution, helping to exclude mimics and provide additional diagnostic information such as the clonality of IgG.^12^^,^^36^^,^^37^

Interestingly, optic nerve involvement could have been demonstrated in 116/201 (58%) patients with MS, whereas optic neuritis was the first clinical event in 76/201 (39%) patients, thus underlining the importance of including objective paraclinical tests to demonstrate involvement. When optic nerve involvement, mainly demonstrated via VEP would not have been considered, 1/201 (0.5%) MS diagnosis would have been missed, due to the high proportion of patients with DIS in ≥4/5 neuroanatomical topographies and of inflammatory CSF changes demonstrated via CSF-restricted oligoclonal bands and KFLC, which constitutes a high-risk population. Although pathological VEP findings were present in a substantially larger proportion of patients, VEP rarely represented the sole diagnostic feature required to establish MS. In patients with DIS in only 2/5 neuroanatomical topographies, inflammatory CSF findings were present in all cases and optic nerve involvement could additionally be demonstrated by MRI in most patients. Consequently, exclusion of VEP could be compensated by other diagnostic criteria in all but one patient. Therefore, VEP retains its high clinical value due to rapid execution, cost-effectiveness, and applicability compared to orbital MRI, which is associated with a longer scan duration. Despite the high proportion of pathological VEP, OCT would also be applicable in a subset of patients to demonstrate optic nerve involvement. Further, OCT is emerging as a robust prognostic biomarker in MS and was also suggested to be applicable for treatment decisions and differential diagnosis.^10^^,^^17^^,^^38^, ^39^, ^40^

The last main finding of the present study is that need for novel MRI markers (CVS, PRL) is limited to a low number of patients, whenever the conventional routine diagnostic as proposed for the 2017-revised McDonald criteria are employed and results are available.^8^^,^^9^ CVS and PRL were needed in only a minority of individuals with CIS and RIS (6.5%) that did not fulfil the diagnostic criteria using DIT or CSF results. Further, PRL could possibly exclusively contributing to diagnosis in only eight cases (4%) in the present study. Furthermore, technical demands in the acquisition process as well as the need for training of neuroradiologists to improve diagnostic certainty limits the availability of these novel MRI markers at present.^20^^,^^32^^,^^33^^,^^41^, ^42^, ^43^ Nevertheless, studies employing the select-6∗ methodology for CVS have reported high sensitivity and specificity for distinguishing MS from mimics, suggesting that wider adoption is of need, but may be limited to dedicated centres and a subgroup of patients.^32^^,^^33^

Limitations of the present study include its single-centre design and the lack of systematic assessment of novel diagnostic markers including CVS, PRL, and OCT, which represent important components of the 2024-revised McDonald criteria. Consequently, the present study primarily evaluates the applicability of the revised criteria using conventional diagnostic tools already established in routine clinical practice (MRI with gadolinium enhancement, VEP, CSF) as proposed for the 2017-revised McDonald criteria. The prospective, real-world design offered follow-up data of individuals with CIS and RIS to assess progression to clinical definite MS. However, follow-up duration was relatively short in some patients. Furthermore, the study was not designed to formally assess diagnostic specificity or rates of potential misclassification, as individuals without suspected inflammatory demyelinating CNS disease were not systematically included. In addition, the study population consisted of individuals referred to a tertiary university hospital, which may limit generalisability to unselected patient populations and to patients presenting in non-specialised settings. Further, gender identity, race and ethnicity were not systematically assessed and therefore sex-specific, gender-related, or race-/ethnicity-associated effects could not be evaluated. Lastly, some individuals with MRI findings suggestive of inflammatory demyelination might potentially fulfil the 2024-revised McDonald criteria but not the currently established RIS criteria and therefore would not have been included in the present study.^15^^,^^16^

In conclusion, application of the routinely available components of the 2024-revised McDonald criteria enhances diagnostic sensitivity and allow for earlier MS diagnosis primarily through inclusion of optic nerve involvement and recognition of high-risk RIS cases. CSF analysis enables diagnosis in a high number of patients, providing both high diagnostic yield and essential differential diagnostic information. While novel imaging markers are promising, their current clinical impact is modest when complete standard MRI and CSF parameters are available. These findings support a pragmatic approach in which new diagnostic tools are integrated into existing, current diagnostic methodologies.

## Contributors

FFK and TS conceptualised the study. FFK, LMA, and ALS developed the methodology. FFK, NM, JR and TS accessed and verified the underlying data. FFK and TS performed the formal analysis. FFK, LMA, and ALS curated the data. FFK and TS wrote the original draft. NM, LMA, GA, ALS, MH, AS, EB, KFJ, SN, LGL, TH, CT, PS, MWH, MP, KWS, MK, CF, AP, SGM, MPW, AH, XM, JR, and TS reviewed and edited the manuscript. TS supervised the study. All authors had full access to the study data relevant to their contributions. All authors read and approved the final version of the manuscript. FFK and TS had final responsibility for the decision to submit for publication.

## Data sharing statement

De-identified individual participant data and a data dictionary defining each field in the dataset will be available upon reasonable request from the corresponding author beginning at the time of publication. Requests for access to the data should be directed to the corresponding author and will be considered by the investigators in consultation with the relevant ethics committees. Data will be shared with researchers whose proposed use of the data has been approved and after signing an appropriate data access agreement.

## Declaration of interests

FFK received honoraria for lectures and travel compensation from Alexion, argenx, Merck, Novartis, and Takeda. He received research support as a fellow of the German Research Foundation (DFG)- and Hannover Medical School (MHH)-funded Clinician Scientist Program (PRACTIS) at MHH and from Merck, Siemens, and the Erwin-Röver Foundation. NM received travel compensation from Penumbra and Sanofi. LMA declares no competing interests. GA reports grants from Instituto de Salud Carlos III (Grant PI22/01570), honoraria for lectures from Roche, Bristol-Myers Squibb, UCB, Centro Universitario de EM (CUEM) del Hospital Ramos Mejía (Buenos Aires), Societat Catalana de Neurologia, and Fundación Santa Fe de Bogotá, support for attending scientific meetings from Novartis, Roche, Bristol-Myers Squibb, ECTRIMS, and EAN, and participation on advisory boards for UCB. ALS declares no competing interests. MH reports personal honoraria for lectures, presentations, and manuscript writing from Roche Pharma, Bayer, Praxis Knabe/Blomberg Hildesheim, and BVA; support for attending meetings and travel from Janssen Cilag, Genentech, Iveric Bio (now Astellas Pharma), Bayer, Théa Pharma, and Merck; and participation on advisory boards for Roche Pharma AG. AS declares no competing interests. EB declares no competing interests. KFJ reports support for attending scientific meetings from Merck, argenx, Novartis, and Neuraxpharm. SN reports personal honoraria for lectures and presentations, as well as travel grants, from argenx, Merck, and Novartis, and support for attending scientific meetings from Merck. She also serves as an unpaid Board Member/Representative of the Young Professionals' Section of the German Society for CSF Diagnostics/Clinical Chemistry (DGLN, Junge Liquorologie). LGL was supported by the PRACTIS Clinician Scientist Program funded by Hannover Medical School and the German Research Foundation (DFG ME 3696/3). TH reports grants from the Petermax-Müller Foundation, honoraria for lectures from argenx, and support for attending scientific meetings from Merck. CT reports payments for expert testimony from Alexion, honoraria for lectures from Dresden International University GmbH, and a leadership role in the Neuromyelitis Optica Study Group. PS declares no competing interests. MWH received research support from Myelitis e.V., the German Federal Joint Committee/Innovation Fund, NEMOS e.V., and argenx; speaker honoraria from selpers og, AMGEN/Horizon, and Alexion; travel grants from Alexion; and compensation for serving on advisory boards from Alexion, Roche, AMGEN, and UCB. MP reports honoraria for lectures from argenx, Alexion, Biogen, Bayer, Demecan, Hexal, Merck, Sanofi, Takeda, Teva, Janssen, Roche, and Novartis, as well as support for attending scientific meetings from Alexion, Biogen, Merck, Sanofi, and Novartis. KWS reports honoraria for lectures and travel reimbursements for attending scientific meetings from Bavarian Nordic, Biogen, Bristol-Myers Squibb, Merck, Mylan, Novartis, Roche, and Viatris, as well as research support from Bristol-Myers Squibb. MPW reports speaker or consultancy honoraria from Bayer Healthcare, Biogen, Biologix, Celgene, Genilac, Imcyse, IXICO, Medison, Merck Serono, Novartis, Roche, Sanofi-Genzyme, Lilly, and Eisai. MK declares no competing interests. CF declares no competing interests. AP reports grant support for remyelination research (RESTORE trial; grant support from a private Dutch foundation for internuclear ophthalmoparesis) and royalties for the chapter “Internuclear Ophthalmoparesis” in UpToDate (Wolters Kluwer). SGM reports grants from the German Research Foundation (DFG), the German Federal Ministry of Food and Agriculture (BfR), the German Multiple Sclerosis Society (DMSG), the Hempel Foundation for Science, Art and Welfare, the Ministry of Culture and Science of the State of North Rhine-Westphalia, and Heinrich Heine University Düsseldorf; honoraria for lectures; and travel support from Academy 2, argenx, Alexion, Almirall, Amicus Therapeutics Germany, Bayer Health Care, Biogen, BioNTech, BMS, Celgene, Datamed, Demecan, Desitin, Diamed, Diaplan, DIU Dresden, DPmed, Gen Medicine and Healthcare Products, Genzyme, Hexal AG, IGES, Impulze GmbH, Janssen Cilag, KW Medipoint, MedDay Pharmaceuticals, Medudy, Merck Serono, MICE, Mylan, Neuraxpharm, Neuropoint, Novartis, Novo Nordisk, ONO Pharma, Oxford PharmaGenesis, QuintilesIMS, Roche, Sanofi-Aventis, Springer Medizin Verlag, STADA, Chugai Pharma, Teva, UCB, Viatris, Wings for Life International, and Xcenda. His research is funded by the German Federal Ministry of Education and Research (BMBF), the Bundesinstitut für Risikobewertung (BfR), the German Research Foundation (DFG), the Else Kröner Fresenius Foundation, the Federal Joint Committee (G-BA), the German Academic Exchange Service, the Hertie Foundation, the Interdisciplinary Centre for Clinical Studies (IZKF) Münster, and the German Foundation Neurology. AH reports honoraria for participation on advisory boards from BMS, Galapagos, Johnson & Johnson, and Sanofi, as well as honoraria for lectures from AbbVie, Alexion, BMS, Kyverna, Medscape, Merck Serono, Neuraxpharm, Pfizer, Sandoz, and Sanofi. XM's institution has received compensation for lecture honoraria and travel expenses, participation in scientific meetings, clinical trial steering committee membership, and clinical advisory board participation in recent years from AbbVie, Actelion, Alexion, Bial PD, Biogen, Bristol-Myers Squibb/Celgene, EMD Serono, Genzyme, Hoffmann-La Roche, Immunic Therapeutics, Janssen Pharmaceuticals, MedDay, Merck, Mylan, Nervgen, Neuraxpharm, Novartis, Peervoice, Samsung Bioepis, Sandoz, Sanofi-Genzyme, Teva Pharmaceuticals, TG Therapeutics, Excemed, Medscape, ECTRIMS, MSIF, and NMSS or their affiliates. JR declares no competing interests. TS reports research support from Alnylam, CSL Behring, Merck, Novartis, and Siemens; and honoraria for lectures, travel support for meeting attendance, and/or consultancy fees from Alexion, Alnylam, Amgen, argenx, Bayer, Biogen, Bristol Myers Squibb, Centogene, CSL Behring, Grifols, Hexal, Horizon, Janssen, Merck, Novartis, Pfizer, Purpose Pharma, Roche, Sanofi, Siemens, SOBI, Teva, and Viatris.

## Appendix A Supplementary data

Supplementary Appendix

Translated abstract
