# Supplementary material for: Applying the 2024-revised McDonald criteria for multiple sclerosis using conventional diagnostic tools: a single-centre prospective cohort study in Germany
Source: eClinicalMedicine. 2026 Jul 25;98:104098. doi: 10.1016/j.eclinm.2026.104098 (PMC13430206; doi:10.1016/j.eclinm.2026.104098)
Supplement: Supplementary Appendix [file mmc1.pdf]

Prospektive Multiple Sklerose Kohorte

Prospektive Kohorten-Studie der Klinik für Neurologie, des Instituts für Neuroradiologie, der Klinik für Psychiatrie und der Klinik für Augenheilkunde bei Patienten mit früher Multipler Sklerose bzw. einem klinisch isolierten Syndrom

STUDIENPROTOKOLL / PRÜFPLAN
Version 1.0 vom 09.12.2019

Inhaltsverzeichnis

STUDIENSYNOPSE/ ZUSAMMENFASSUNG 2

VERANTWORTLICHKEITEN 2

ZIELSETZUNG UND BEGRÜNDUNG DER STUDIE 2

CHARAKTERISIERUNG DER PATIENTEN 3

EINSCHLUSSKRITERIEN 4

AUSSCHLUSSKRITERIEN 4

STUDIENTYP 4

CHARAKTERISIERUNG DER BEABSICHTIGTEN MAßNAHMEN 4

AUSFÜHRLICHE BESCHREIBUNG DES STUDIENABLAUFS 8

VERLAUFSUNTERSUCHUNGEN 9

ZIELGRÖßEN 9

DATENERFASSUNG UND DOKUMENTATION 9

UNERWÜNSCHTE EREIGNISSE 10

AUSWERTUNG/BIOMETRIE 10

UNTERSCHRIFTEN 11

Studiensynopse / Zusammenfassung

s. Dokument Studiensynopse

Verantwortlichkeiten

Die Studie wird von Herrn Prof. Dr. med. Thomas Skripuletz als verantwortlichem Projektleiter der Klinik für Neurologie (OE 7210, Carl-Neuberg-Str. 1, 30625 Hannover) und Herrn Prof. Dr. Dr. Mike P. Wattjes als verantwortlichem Projektleiter des Instituts für Diagnostische und Interventionelle Neuroradiologie (OE 8210, Carl-Neuberg-Str. 1, 30625 Hannover) durchgeführt. Alle genannten Personen tragen dabei die Verantwortung, die Studie nach folgendem Prüfplan durchzuführen und die Richtigkeit und Validität der erhobenen Daten nach bestem Wissen und Gewissen sicher zu stellen.

Zielsetzung und Begründung der Studie

Die Multiple Sklerose (MS) ist die häufigste chronisch entzündliche demyelinisierende Erkrankung des Zentralen Nervensystems bei jungen Erwachsenen mit einem erhöhten Risiko einer Langzeitbehinderung. Für die Diagnose einer MS spielt neben der klinisch neurologischen Untersuchung und der Liquoruntersuchung (Nachweis liquorspezifischer oligoklonaler Banden) die Magnetresonanztomographie (MRT) des Gehirns und des Rückenmarks eine entscheidende Rolle. Mit Einführung der 2010 McDonald Kriterien ist es mit Hilfe der MRT möglich, die MS bereits nach dem ersten klinischen Ereignis zu diagnostizieren, welches eine sehr frühzeitige Diagnose und somit auch eine frühzeitige Behandlung ermöglicht.

Obgleich das optische System, insbesondere der Sehnerv, eines der häufigsten (Erst)Manifestationen der MS darstellt, sind Läsionen im Sehnerv (auf Basis von MRT oder optischer Kohärenztomographie (OCT)) bisher nicht in den Diagnosekriterien aufgenommen worden. Die MAGNIMS Gruppe hat kürzlich alternative MS Diagnosekriterien vorgeschlagen, welche Läsionen innerhalb des Sehnervs für den Nachweis einer örtlichen Dissemination der MS Krankheitsaktivität beinhaltet. Dieser Vorschlag wurde sehr kontrovers diskutiert und ist letztendlich nicht in die aktuelle 2017 publizierte Revision der McDonald Kriterien aufgenommen worden. Ein wesentlicher Grund war die Tatsache, dass eine standardisierte Bildgebung des optischen Systems, insbesondere des Sehnervs, schwierig zu implementieren

ist.

Eine standardisierte und umfassende Charakterisierung der MS bzw. CIS Patienten ist von besonderer Relevanz. Es gibt zunehmende Evidenz, dass bestimmte klinische und neuroradiologische Parameter zu Beginn der Erkrankung einen hohen Prädiktiven Wert für die mittelfristige und langfristige Prognose der Patienten hat. Bisherige Kohortenstudien beinhalten eine unzureichende Phänotypisierung der Patienten, wobei häufig das spinale MRT, die Bildgebung des Sehnervs sowie die Liquoruntersuchung unvollständig sind.

Wichtige Zielsetzungen dieser prospektiven Studie sind:

- Untersuchung des Nutzens von Bildgebung des optischen Systems (Sehnerven) auf gegenwärtige und alternative MS Diagnosekriterien.
- Untersuchung des Nutzens alternativer MRT Pulssequenzen (Double Inversion recovery) u.a. zum Nachweis von Läsionen im Nervus opticus.
- Identifikation von prädiktiven Parametern (MRT, Liquor, Neurophysiologie, OCT etc.) hinsichtlich des Krankheitsverlaufs und der Krankheitsprogression.
- Untersuchung der Sensitivität der MRT Bildgebung im Vergleich zur Neurophysiologie und OCT hinsichtlich der Detektion von Läsionen im optischen System.
- Untersuchung neuer MRT Parameter für Inflammation („leptomenigeal enhancement“) und Progression („smoldering lesions“).

Charakterisierung der Patienten

Das Kollektiv besteht aus männlichen und weiblichen Patienten, die sich mit neuen Symptomen in unserer Klinik vorstellen hinweisend auf eine MS. Insgesamt sollen etwa 400 Patienten rekrutiert werden. Als Kontrollen dienen Patienten, die sich mit Symptomen hinweisend auf eine MS vorstellen und untersucht werden, bei denen jedoch eine MS oder CIS nicht diagnostiziert werden. Es wird eine Fallzahl von 200 Patienten mit einer frühen MS bzw. CIS und 200 Kontrollpersonen angestrebt. Wir antizipieren eine jährliche Rekrutierung von 50 Patienten mit einer MS bzw. CIS pro Jahr, so dass der Einschlusszeitraum 4 Jahre beträgt. Eingeschlossen werden dabei Patienten, die sich zwischen dem 01.02.2020 und 01.02.2022 in der neurologischen Klinik vorstellen. Der Einschluss erfolgt nur nach Erfüllen der unten aufgelisteten Einschlusskriterien, sowie nach Abklärung der üblichen

Kontraindikationen für die MRT Bildgebung. Es ergibt sich keine geldliche Vergütung oder Entschädigung für die teilnehmenden Patienten.

Einschlusskriterien

Einschlusskriterien sind jeweils die Volljährigkeit und Mündigkeit sowie das Vorliegen einer schriftlichen Einwilligung nach entsprechender Aufklärung. Voraussetzung für den Einschluss in die Studie ist zudem das Vorliegen eines ersten klinischen Syndroms hinweisend auf eine demyelinisierende Erkrankung des zentralen Nervenystems (MS oder CIS). Patienten, bei denen sich im Verlauf die Diagnose einer MS oder eines CIS nicht bestätigt, werden nachträglich als Kontrollen beurteilt. Ausschlusskriterien sind Patienten mit einer eingeschränkten Kommunikationsfähigkeit, bei denen die Gespräche nur eingeschränkt möglich sein werden.

Ausschlusskriterien

Ausschlusskriterien sind Patienten mit einer Kontraindikation für die Durchführung einer MRT Untersuchung (z.B. Herzschrittmacher, MRT-inkompatible Implantate, Klaustrophobie, allergische Reaktionen gegen Gadolinium (Gd)-basierte Kontrastmittel). Weitere Ausschlusskriterien sind Patienten mit präexistenten neurodegenerativen, vaskulären, neoplastischen oder entzündlichen Erkrankungen (einschließlich des optischen Systems).

Studientyp

Beobachtungsstudie mit prospektivem Einschluss, monozentrisch, offen.

Charakterisierung der beabsichtigten Maßnahmen

Patienten, bei denen klinisch der dringende Verdacht auf eine MS oder ein CIS besteht werden über die Studie aufgeklärt und nach Einwilligung in die Studie aufgenommen. Die geplanten Maßnahmen sind im Einzelnen:

1. Strukturierte klinische Untersuchung inkl. neurologischer Befunderhebung.
2. Die zerebrale und spinale Bildgebung mittels MRT mit Kontrastmittel. Die MRT vom Kopf wird nach Maßgabe der üblichen Kontraindikationen (Metallimplantate,

Platzangst, Herzschrittmacher, Prothesen), sowie Kontraindikationen zum Kontrastmittel (Allergie bekannt, drastisch reduzierte Nierenfunktion) durchgeführt. Es sind folgende Bildakquisitionsparameter an einem whole-body MRT zur Darstellung und Charakterisierung eventueller zerebraler und spinaler Läsionen vorgesehen:

Zerebrale Bildgebung (einschließlich des Sehnervs) bei 3 Tesla mit i.v. Gd. basiertes Kontrastmittel

- 3D T1 gradienten echo
- Suszeptibilitätsgewichtete Sequenzen
- Diffusionstensor Bildgebeung
- Whole brain Spektroskopie
- Axiale T1 (turbo)spin echo
- Axiale T2 (turbo)spin echo
- Coronale T2 durch Orbita/Sehnerv mit Fettsättigung
- 3D FLAIR
- 3D DIR
- Axiale T1 (turbo)spin echo i.v. Kontrast
- Coronale T1 durch Orbita/Sehnerv mit Fettsättigung

(Untersuchungszeit ca. 60 Minuten)

Spinale Bildgebung bei 1,5 oder 3 Tesla mit i.v. Gd. basiertes Kontrastmittel

- Sagittale T1 (turbo)spin echo
- Sagittale short tau inversion recovery (STIR)
- Sagittale T2 (turbo)spin echo
- Axiale T2 (turbo)spin echo

(Untersuchungszeit ca. 30 Minuten)

Die Kontrastdosis makrozyklischer Gd-Kontrastmittel für die zerebrale und evtl. spinale Bildgebung erfolgt mit einer Standarddosis (0,1 mmol/kg/Körpergewicht), welche für die reguläre Krankenversorgung verwendet wird.

3. Blutentnahme (3 Serumröhrchen und 2 große EDTA-Röhrchen) über eine liegende periphere Venenverweilkanüle. Die Analyse des Blutserums dient v.a. der Beurteilung einer etwaigen Störung der Blut-Liquorschranke bzw. einer möglichen intrathekalen Immunglobulin-Synthese. Aus dem EDTA-Blut werden die peripheren mononukleären Blutzellen isoliert und anschließend analysiert. Im Rahmen der Routinediagnostik bei Patienten mit Verdacht auf eine MS/CIS erfolgt nach Aufklärung und Einwilligung eine Lumbalpunktion, um Liquor zu gewinnen. Anschließend erfolgt eine Analyse des Liquors im Hinblick auf Zellzahl, Differentialzellbild, Gesamtprotein, Serum-/Liquor-Albuminquotient, intrathekale Immunglobulinsynthese und Vorliegen oligoklonaler Banden. Ein Teil des Liquors wird im Institut für experimentelle Infektionsforschung des Twincore mittels Massenspektrometrie auf mögliche Biomarker hin untersucht werden. Im Verlauf können ggf. weitere Techniken wie z.B. durchflusszytometrische Analysen (FACS-Analysen) der Immunzellen des Blutes oder Untersuchungen hinsichtlich der Expression bestimmter Chemokine bzw. Zytokine (bspw. IL-17, IL-1, IL-6) im Liquor mittels ELISA-Technik erwogen werden.

4. Neuropsychologie und psychiatrische Untersuchung. Die geplante nicht invasive neuropsychologische Testung teilt sich in zwei Abschnitte, die beide Teil der implementierten Routinediagnostik an unserer Klinik sind.
   a. Consortium to Establish a Registry for Alzheimer's Disease (CERAD)-PLUS
      Die CERAD PLUS-Testbatterie besteht aus acht Aufgaben. Der Proband wird dazu aufgefordert Figuren zu zeichnen, sich an Wörter zu erinnern sowie Wörter wieder zu erkennen und Dinge zu benennen. Des Weiteren sollen Buchstaben und Nummern in der richtigen Reihenfolge verbunden werden (Trail making Test) und zu zwei Kategorien wie S-Wörter oder Tiere so viele Begriffe aufgeschrieben werden wie möglich (Phonematische Flüssigkeit). Die Testung erfolgte hierbei nach Schmid NS et al. (2014).
      (Untersuchungszeit ca. 50 Minuten)
   b. *Die Testbatterie zur Aufmerksamkeitsprüfung (TAP)*
      Aus der computergestützten TAP werden die vier Teilaspekte Alertness, geteilte Aufmerksamkeit, Arbeitsgedächtnis und Daueraufmerksamkeit durchgeführt.
      Beim „Alertness“-Test wird die Reaktionsgeschwindigkeit des Probanden auf

einen Reiz mit und ohne Warnton in vier Durchläufen getestet, wobei die Testzeit ca. 4,5 min beträgt und die Reaktion einen Tastendruck darstellt.

Der Untertest „Geteilte Aufmerksamkeit" besteht aus zwei Aufgabenteilen, die gleichzeitig durchgeführt werden sollen. Die eine Aufgabe beinhaltet ein bestimmtes visuelles Muster und die zweite Aufgabe ein auditives Muster, auf das mittels Betätigung einer Taste reagiert werden soll.
(Untersuchungszeit ca. 5 Minuten)

Das Arbeitsgedächtnis wird mithilfe von Zahlenfolgen geprüft. Der Proband wird je nach Schwierigkeitsgrad dazu aufgefordert eine Taste zu betätigen, wenn eine Zahl der letzten oder vorletzten gezeigten Zahl gleicht.
(Untersuchungszeit ca. 5 Minuten)

Der Test zur Daueraufmerksamkeit ähnelt dem Test zum Arbeitsgedächtnis. Hier wird eine Folge von Figuren unterschiedlicher Form und Färbung abgebildet. Der Proband wird dazu aufgefordert so schnell wie möglich mittels Tastenbetätigung zu reagieren, wenn sich zwei Figuren in ihrer Form gleichen (Schwierigkeitsgrad 1) oder wenn sie sich in Farbe oder Form gleichen (Schwierigkeitsstufe 2).
(Untersuchungszeit ca. 15 Minuten)
QUELLE: https://www.psytest.net/index.php?page=TAP-2-2&hl=de_DE

Die gemessenen Werte werden in Form von prozentual zu Normwerten der Standartbevölkerung (für den jeweiligen Test) evaluiert. Zusätzlich erfolgt eine standardisierte psychiatrische Untersuchung, um mögliche psychiatrische Komorbiditäten festzustellen bzw. auszuschließen.

5. Augenärztliche Untersuchungen.

- Visusprüfung (BCVA= Best corrected Visual Acuity). Hierbei handelt es sich um eine Visusprüfung anhand einer objektiven Refraktion. Es wird zum Einen der Visus bei hohem Kontrast und zum Anderen der für Veränderungen des Sehnerven sensitiveren low contrast Visus bestimmt.

(Untersuchungszeit ca. 15 Minuten)

- Gesichtsfeldperimetrie. Bei der Perimetrie handelt es sich um eine 30°/10° Gesichtsfelduntersuchung mit dem Octopus 900 von Haag Streit. Es handelt sich um eine statische Perimetrie, bei der das 30°/10° Gesichtsfeld bzw. Ausfälle in diesem ermittelt werden können. Die Untersuchung wird an beiden Augen separat durchgeführt.
  (Untersuchungszeit ca. 20 Minuten)
- Optische Kohärenztomographie (mit OCT-Angiographie). Bei der optischen Kohärenztomographie handelt es sich um ein nicht – invasives Verfahren, um die einzelnen Netzhautschichten darzustellen. Hierbei wird mit Licht die Netzhaut abgetastet und je nach Schicht unterschiedlich zurückreflektiert, sodass ein Bild entstehen kann. Hierbei ist es nicht nur möglich die Makula zu begutachten, sondern die optische Kohärenztomographie bietet auch die Möglichkeit, den Sehnerven und eine Veränderung an diesem zu beurteilen. Bei der anschließenden OCT Angiographie werden die Erythrozyten in den Aderhaut- und Netzhautgefäßen abgetastet und deren „flow" erfasst. Des Weiteren wird mittels Messung der durch Gefäße entstehenden Schatten der Arterio-venöse Quotient bei der Untersuchung mittels optischer Kohärenztomographie gemessen.
  (Untersuchungszeit ca. 10 Minuten)

Alle genannten Maßnahmen sind Teil der Routine Untersuchungen und verursachen kein zusätzliches Risiko für den Patienten.

Ausführliche Beschreibung des Studienablaufs

Geplant ist nach Prüfung der Ein-und Ausschlusskriterien die prospektive Rekrutierung von Patienten, die unter der (Verdachts-)Diagnose einer MS oder eines CIS in der Klinik für Neurologie der MHH zur stationären Diagnostik aufgenommen werden. Der Rekrutierungszeitraum ist dabei zunächst begrenzt auf 4 Jahre. Nach mündlicher Aufklärung über die Bedeutung und das Ziel der Studie, sowie Aushändigung des Aufklärungsbogens, ist das Vorliegen einer schriftlichen Einwilligungserklärung Grundvoraussetzung für den Studieneinschluss. Anschließend wird im Rahmen der

Erstuntersuchung eine strukturierte, klinisch-neurologische körperliche Untersuchung erfolgen. Hiernach sollen die neuropsychologischen, neurophysiologischen, augenärztlichen sowie die MRT Untersuchungen durchgeführt werden. Die Teilnahme ist an keine finanzielle Entschädigung geknüpft. Es besteht ein jederzeitiges Rücktrittsrecht ohne Angabe von Gründen.

Verlaufsuntersuchungen

Die Verlaufsuntersuchungen der Probanden erfolgen zu den Zeitpunkten Jahr 1, Jahr 5 und Jahr 10. Zu diesen Zeitpunkten erfolgen ein zerebrales sowie spinales MRT (ohne i.v. Kontrastmittel, bei entsprechender klinischer Symptomatik kann eine i.v. Kontrastgabe indiziert sein), eine neurologische und neuropsychologisch psychiatrische Untersuchung sowie eine augenärztliche Untersuchung. Geplant ist zudem eine Blutentnahme (3 Serumröhrchen und 2 große EDTA-Röhrchen).

Zielgrößen

Primäres Outcome ist die systematische Analyse der MRT-Parameter bei Patienten mit diagnostizierter MS bzw. CIS. Als sekundäres Outcome wird eine vergleichende Darstellung der untersuchten Parameter in Kontrollpatienten angestrebt. Zudem sollen die strukturiert ermittelten Ergebnisse der augenärztlichen Untersuchungen, neuropsychologischen Untersuchungen, laborchemischen Untersuchungen mit klinischen (Outcome)-Parametern korreliert werden. Des Weiteren sollen mit Hilfe von Screeningmethoden wie der Massenspektrometrie neue Biomarker hinsichtlich Diagnosestellung und Prognoseabschätzung im Liquor von MS Patienten identifiziert werden.

Datenerfassung und Dokumentation

Die im Rahmen der Studie gewonnenen medizinischen Daten werden entsprechend der Datenschutzrichtlinien und unter Berücksichtigung der ärztlichen Schweigepflicht in pseudonymisierter Form aufbewahrt. Die Zuordnung zum Pseudonym erfolgt mittels einer Liste, welche gegen unautorisierten Zugriff geschützt ist. In einem Studienordner

werden die Einwilligungs-, Aufklärungsbögen archiviert. Die Auswertung erfolgt in anonymisierter Form.

Unerwünschte Ereignisse

Die MRT-Untersuchungen sind nach sorgfältiger Prüfung der Kontraindikationen risikoarme Verfahren, welche in der Routinekrankenversorgung dieser Patienten bereits fest etabliert sind. Bei unerwarteter Platzangst bzw. Unruhe oder Lagerungsschwierigkeiten wird die Untersuchung durch die zuständigen technischen Assistenten nicht weiter durchgeführt. Bei allergischer Reaktion auf das Kontrastmittel wird ebenfalls unverzüglich und nach üblicher Handhabe eine anti-anaphylaktische Therapie eingeleitet und eine kardiopulmonale Stabilisierung sichergestellt. Blutentnahme und Liquorgewinnung werden jeweils im Rahmen der Standardroutine Abklärung der Patienten durchgeführt. Zur Liquorgewinnung ist keine zusätzliche Maßnahme, die nicht Teil der Standarduntersuchung ist, notwendig. Die Blutentnahme erfolgt über einen vorhandenen peripheren Venenzugang. Die zu erwartenden unerwünschten Ereignisse decken sich daher mit den grundsätzlichen Eingriffsrisiken. Da kognitive Fähigkeiten nicht invasiv getestet werden, sind keine unerwünschten Ereignisse zu erwarten. Die augenärztlichen Untersuchungen sind Teil der ophthalmologischen Routinediagnostik und werden standardisiert durchgeführt. Es sind keine relevanten Risiken für die Studienteilnehmer zu erwarten. Leichte Irritationen der Hornhaut sind möglich, können jedoch mit benetzenden Augentropfen schnell behandelt werden.

Auswertung/Biometrie

Geplant ist die strukturierte Analyse von MRT-Ergebnissen bei Patienten mit diagnostizierter MS bzw. CIS. Zudem sollen die strukturiert ermittelten Ergebnisse der augenärztlichen Untersuchungen, neuropsychologischen Untersuchungen, laborchemischen Untersuchungen mit klinischen (Outcome)-Parametern korreliert werden. Bei einer Fallzahl von 200 Probanden mit einer MS/CIS soll eine deskriptive Statistik erfolgen. Gruppenvergleiche für parametrische Daten werden mittels T-Tests, für nicht-parametrische Daten werden mittels Mann-Whitney-U-Test erfolgen.

Widerrufsrecht und Weitergabe von Daten und Bioproben

Die Studienteilnehmer haben jederzeit die Möglichkeit, ihre Einwilligung zur Studienteilnahme ohne Angabe von Gründen und ohne erwartbare Nachteile für die laufende Behandlung zurückzuziehen. Im Falle eines solchen Widerrufs können sie entscheiden, ob Ihre Biomaterialien vernichtet und die dazu gehörenden Daten gelöscht werden sollen, oder ob die bereits erhobene Daten und gesammelten Materialien für weitere Forschungsvorhaben verwendet werden dürfen. Anonym gespeicherte, analysierte oder bereits publizierte Daten können nicht nachträglich gelöscht werden, da eine Zuordnung zur Person zu diesem Zeitpunkt nicht mehr möglich ist.

Eine Weitergabe der personenbezogenen Daten an unberechtigte Dritte, etwa Versicherungsunternehmen oder Arbeitgeber, erfolgt nicht. Pseudonymisierte Daten, die an berechtigte Dritte innerhalb der Europäischen Union (EU) weitergegeben werden, dürfen nur für einen beantragten Forschungszweck verwendet und vom Empfänger nicht zu anderen Zwecken weitergegeben werden. Bei pseudonymisierten Daten ist das Identifikationsmerkmal wie Name und Anschrift durch ein Kennzeichen – z.B. eine Codenummer – ersetzt, so dass eine Zuordnung zu einer Person nur über weitere Hilfsmittel – etwa eine Referenzliste – möglich ist.

Unterschriften

Hiermit bestätigen wir, die Studie entsprechend des Protokolls durchzuführen. Die verantwortlichen Projektleiter wissen, dass auch bei einer positiven Beurteilung des Vorhabens durch die Ethikkommission der Medizinischen Hochschule Hannover die ärztliche und juristische Verantwortung uneingeschränkt bei den Projektleitern und ihren Mitarbeitern verbleibt.

Hannover, den

Prof. Dr. med. Thomas Skripuletz

verantwortlicher Projektleiter der Klinik für Neurologie

Prof. Dr. Dr. Mike P. Wattjes

verantwortlicher Projektleiter des Instituts für Diagnostische und Interventionelle Neuroradiologie
