# Supplementary material for: Applying the 2024-revised McDonald criteria for multiple sclerosis using conventional diagnostic tools: a single-centre prospective cohort study in Germany
Source: eClinicalMedicine. 2026 Jul 25;98:104098. doi: 10.1016/j.eclinm.2026.104098 (PMC13430206; doi:10.1016/j.eclinm.2026.104098)
Supplement: Translated abstract [file mmc2.docx]

**Translated abstract**

*The following translations in German were submitted by the authors and we reproduce them as supplied. They have not been peer reviewed. Our editorial processes have only been applied to the original abstract in English, which should serve as reference for this manuscript.*

**Hintergrund**

Die 2024 revidierten McDonald-Kriterien schlagen mehrere Änderungen vor, die eine frühere und zugleich präzisere Diagnose der Multiplen Sklerose (MS) ermöglichen könnten. Zu den Neuerungen gehören die Einbeziehung des Sehnervs als fünfte neuroanatomische Topographie, zusätzliche MRT-Biomarker, Kappa-freie Leichtketten (KFLC) im Liquor sowie die mögliche Einstufung eines radiologisch isolierten Syndroms (RIS) als MS. Die Anwendbarkeit dieser Änderungen in der klinischen Routine bedarf einer weiteren Evaluation.

**Methoden**

In dieser prospektiven Studie wurden nach dem Screening 215 von 255 Personen mit einem ersten demyelinisierenden Ereignis oder einem RIS in die Endanalyse eingeschlossen. Alle Teilnehmenden erhielten eine konventionelle Diagnostik gemäß den 2017 revidierten McDonald-Kriterien, einschließlich kontrastmittelgestützter MRT, visuell evozierter Potenziale (VEP) und Liquordiagnostik. Neu eingeführte MRT-Marker sowie die optische Kohärenztomographie (OCT) standen hingegen nicht systematisch zur Verfügung. Anschließend erfolgte eine retrospektive Reklassifikation gemäß den 2024 revidierten McDonald-Kriterien.

**Ergebnisse**

Bei Anwendung der 2024 revidierten Kriterien erhielten 201 von 215 Personen (93,5%) die Diagnose einer MS, verglichen mit 181 von 215 Personen (84%) nach der Revision von 2017 (p<0,0001). Die zusätzlichen Diagnosen resultierten hauptsächlich aus der Berücksichtigung einer Sehnervbeteiligung (55%) sowie der Reklassifikation eines RIS (35%). Mittels VEP konnten Sehnervläsionen in 89% der Fälle nachgewiesen werden. Insgesamt wurden 102 von 201 Personen (51%) allein anhand der MRT-Befunde diagnostiziert (räumliche Dissemination ≥ 4 Topographien), während bei 99 von 201 Personen (49%) zusätzlich entzündliche Liquorbefunde und eine zeitliche Dissemination berücksichtigt wurden.

**Interpretation**

In dieser Real-World-Kohorte führte die Anwendung ausgewählter Modifikationen der 2024 revidierten McDonald-Kriterien zu einer höheren diagnostischen Sensitivität, vor allem durch die Einbeziehung der Sehnervbeteiligung und die Berücksichtigung eines RIS. Bei den meisten Betroffenen war eine MS-Diagnose möglich. Die Liquordiagnostik blieb sowohl für die Diagnosestellung als auch für die Differentialdiagnostik von zentraler Bedeutung. Zusätzliche MRT-Biomarker könnten die diagnostische Sensitivität künftig weiter erhöhen.

**Finanzierung**

Es wurden keine externen Fördermittel für diese Studie eingeworben.
